# High intensities of population movement were associated with high incidence of COVID-19 during the pandemic

**DOI:** 10.1017/S0950268820001703

**Published:** 2020-08-03

**Authors:** Huikuan Yang, Dandan Chen, Qunfang Jiang, Zhaohu Yuan

**Affiliations:** 1 Department of Blood Transfusion, Guangzhou First People's Hospital, School of Medicine, South China University of Technology, Guangzhou 510180, Guangdong, China; 2 Department of Radiology, Guangzhou First People's Hospital, School of Medicine, South China University of Technology, Guangzhou 510180, Guangdong, China; 3 Department of Clinical Laboratory, Guilin Women's and Children's Hospital, Guilin 541001, Guangxi, China

**Keywords:** COVID-19, intracity travel intensity, population movement, SARS-CoV-2

## Abstract

Increased population movements and increased mobility made it possible for severe acute respiratory syndrome coronavirus 2, which is mainly spread by respiratory droplets, to spread faster and more easily. This study tracked and analysed the development of the coronavirus 2019 (COVID-19) outbreak in the top 100 cities that were destinations for people who left Wuhan before the city entered lockdown. Data were collected from the top 100 destination cities for people who travelled from Wuhan before the lockdown, the proportion of people travelling into each city, the intensity of intracity travel and the daily reports of COVID-19. The proportion of the population that travelled from Wuhan to each city from 10 January 2020 to 24 January 2020, was positively correlated with and had a significant linear relationship with the cumulative number of confirmed cases of COVID-19 in each city after 24 January (all *P* < 0.01). After the State Council launched a multidepartment joint prevention and control effort on 22 January 2020 and compared with data collected on 18 February, the average intracity travel intensity of the aforementioned 100 cities decreased by 60−70% (all *P* < 0.001). The average intensity of intracity travel on the *n*th day in these cities during the development of the outbreak was positively related to the growth rate of the number of confirmed COVID-19 cases on the *n* + 5th day in these cities and had a significant linear relationship (*P* < 0.01). Higher intensities of population movement were associated with a higher incidence of COVID-19 during the pandemic. Restrictions on population movement can effectively curb the development of an outbreak.

## Introduction

Severe acute respiratory syndrome coronavirus 2 (SARS-CoV-2) is a single-stranded, positive-sense RNA betacoronavirus mainly enveloped by respiratory droplets that was first reported in Wuhan, China, in December 2019 and has had an enormous impact in China and worldwide. The disease caused by SARS-CoV-2 is called coronavirus disease 2019 (COVID-19) [1, 2]. Since the outbreak of COVID-19 in Wuhan in December 2019, as of 14 April 2020, SARS-CoV-2 had rapidly spread to more than 200 countries worldwide, causing 1 776 867 people to become infected and 111 828 to die [3, 4]. Because SARS-CoV-2 has high infectivity and causes high mortality, it has aroused great public health concerns [5, 6]. On 11 March 2020, COVID-19 was classified as a pandemic by the World Health Organization (WHO) [6].

Wuhan is a large provincial capital city with a population of 12.00 million. It is also one of China's most important bases of industry, science and education, as well as a major transportation hub [7]. As the geographic centre of China, Wuhan is known as the ‘major juncture of nine provinces’. It is the largest land, water and air transportation hub in China and provides a shipping centre in the middle reaches of the Yangtze River. Its high-speed rail network radiates to more than half of China, and it is the only city in Central China with direct access to five continents [7]. Wuhan was also the first city in China where SARS-CoV-2 was transmitted and where it infected most people [8, 9]. The number of confirmed cases in Wuhan accounted for 59.71% (50 008/83 745) of the total number of confirmed cases in China, and the number of deaths accounted for 76.94% (2579/3352) of the total deaths in China [9]. The occurrence of the SARS-CoV-2 outbreak in Wuhan coincided with China's major traditional festival – the Spring Festival. Wuhan is a city where many people travel from elsewhere. It is very common for students and migrant workers to return home for the Spring Festival (Chinese Lunar New Year). Due to the impact of the Spring Festival and the epidemic conditions, approximately five million people left Wuhan on the eve of the Spring Festival [10]. This aroused great public concern about which cities these five million people travelled to and how they affected the development of the epidemic situation in those cities. Some reports indicated that the population moving out of Wuhan mainly migrated to other cities in Hubei Province and to some large and medium-sized cities in China [11].

To control the COVID-19 epidemic more effectively, on 22 January 2020, the Party Central Committee and the State Council launched a multidepartment joint prevention and control mechanism. The next day, Wuhan announced the lockdown of the city [12–14]. After that, Hubei and the rest of China adopted unprecedented measures to prevent and control the epidemic. These measures included large-scale quarantine and isolation, extensive monitoring of suspected cases and strict population movement control [13, 14]. This study used big data provided by Baidu Maps Smarteye to track and analyse the epidemic situation in the top 100 cities that were the destinations of people who travelled out of Wuhan from 10 to 24 January 2020. This study also clarified the correlation between the epidemic situation and the number of people travelling out of Wuhan. Meanwhile, we explored the changes in intracity travel intensity in these cities after strictly controlling the movement of people and its effects on the *n*th day in these cities on the growth rate of the number of confirmed COVID-19 cases on the *n* + 5th day.

## Methods

### Data sources

This study was mainly based on two data sources. The first was the number of newly confirmed cases, cumulative confirmed cases, recovered cases and fatal cases of COVID-19 reported daily by municipal, provincial and national health committees (confirmed based on real-time RT-PCR testing) [15]. Second, the Baidu Maps Smarteye database provided the top 100 cities that were the first destination cities of people travelling out of Wuhan, the proportion of people travelling into Wuhan from each city from 10 January 2020 to 24 January 2020 (reflecting the size of the population travelling out of and into Wuhan and showing the horizontal comparison among cities) and intracity travel intensity in the 100 cities during the period from 18 January 2020 to 17 February 2020. The intracity travel intensity was the index result of the ratio of the number of people who travelled in the city to the population of the city [11].

### Study design

During the outbreak of COVID-19, most cities in China adopted strict control over population movement to control the development of COVID-19 [12–14]. In this study, we explored the correlation between population movement and the epidemic situation. The average incubation period of SARS-CoV-2 is 5.2 days (95% CI 4.1–7.0) [5]. Thus, for this research study, the average incubation period was calculated as five days. We explored the effect of intracity travel intensity on the *n*th day in the top 100 cities on the growth rate of the number of confirmed COVID-19 cases on the *n* + 5th day. The growth rate of confirmed cases = the number of newly confirmed cases × 100/the number of existing confirmed cases and the number of existing confirmed cases = the cumulative confirmed cases – the cumulative cured cases – the cumulative deaths.

### Graphs and statistical analysis

All graphs were generated using Prism 4 (GraphPad Software, Inc., California, USA). Statistical significance was assessed using bivariate correlation and linear regression (*P* < 0.05 was considered significant) in SPSS 22.0 (SPSS, Inc., Chicago, IL, USA). The columns are the mean of the triplicate experiments (bars ± s.d.; *P < 0.05, **P < 0.01).

## Results

### Analysis of the destinations of the people travelling from Wuhan

**Fig. 1.**
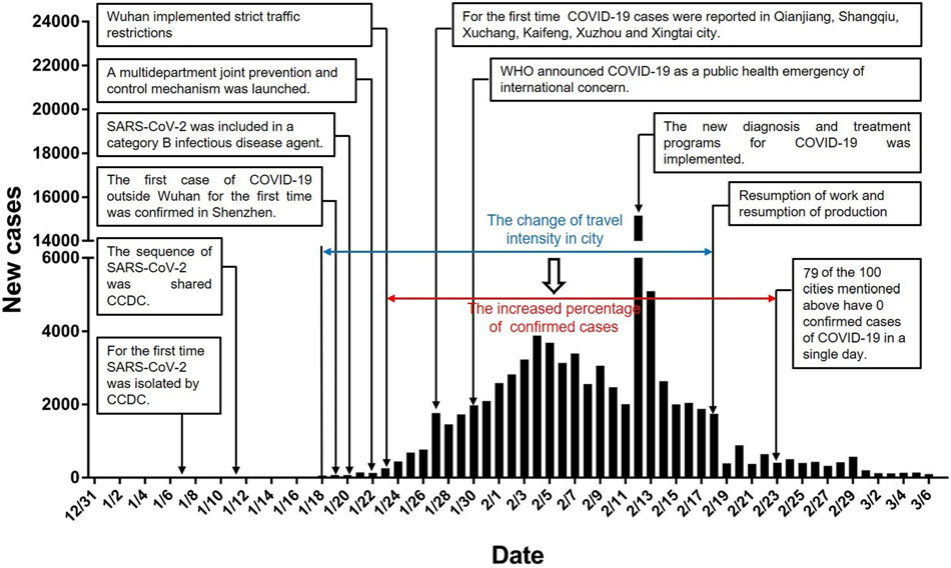
Timeline of key SARS-CoV-2 events and new cases by day in China.

**Fig. 2.**
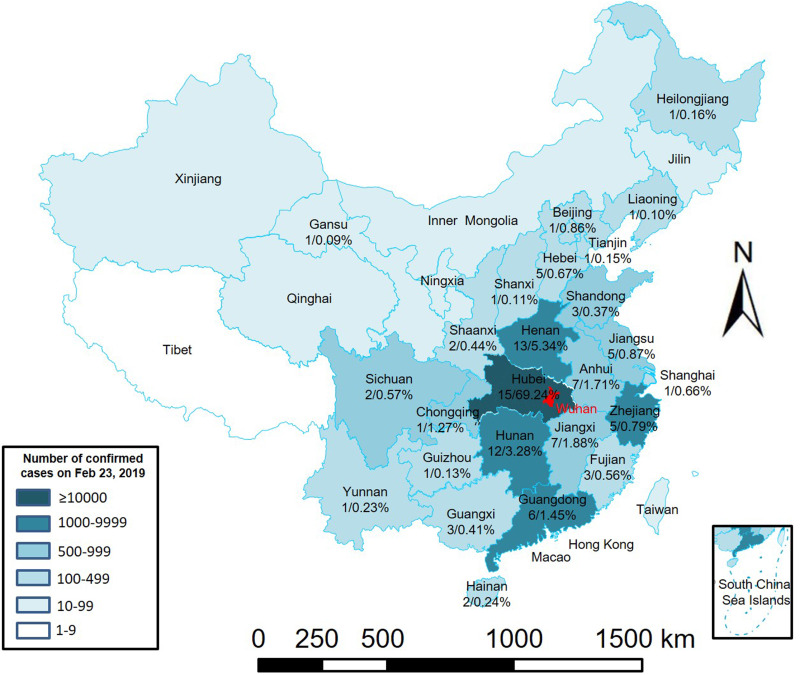
Proportion of the population travelling out of Wuhan to various provinces and cities from 10 January 2020 to 24 January 2020. The provinces where the top 100 cities are located and cumulative confirmed number of COVID-19 cases in each city on 23 February 2020. The numbers at the bottom of each province represent the number of cities out of the top 100 cities and the percentage of travellers who left Wuhan in this province.

Figure 1 shows China's epidemic development flowchart. From 31 December 2019 to 23 January 2020, due to the Spring Festival and epidemic factors, approximately five million people left Wuhan [10]. From 10 January 2020 to 24 February 2020 (considering that Wuhan was closed on 23 January, and only special staff left the city on the 24th), the data provided by Baidu Maps Smarteye show that among the top 100 destination cities for people who left Wuhan, 15 were in Hubei Province, and the population moving to those cities accounted for 68.24% of the people who left Wuhan. Most went to Xiaogan, Huanggang and Jingzhou, which accounted for 13.80%, 13.04% and 6.54%, respectively, of people who left Wuhan. Outside of Hubei, Henan, Hunan and Jiangxi Provinces received the most people from Wuhan (Fig. 2 and Table 1). Among the top 100 destination cities for people who left Wuhan, 13 were in Henan, and the population moving to Henan accounted for 5.34% of people who left Wuhan. In Henan Province, most went to Xinyang, Nanyang and Zhumadian, accounting for 1.49%, 0.69%, and 0.66%, respectively, of those who left Wuhan. Among the top 100 destination cities for people who left Wuhan, 12 were in Hunan, and the population moving to Hunan accounted for 3.28% of people who left Wuhan. Most went to Changsha, Yueyang and Changde in Hunan Province, accounting for 1.02%, 0.52% and 0.33% of the people who left Wuhan, respectively. Among the top 100 destination cities for people who left Wuhan, seven were in Jiangxi Province, and the population moving to Jiangxi accounted for 1.88% of the people who left Wuhan. Most went to Jiujiang, Nanchang and Yichun, accounting for 0.52%, 0.48% and 0.26%, respectively, of the people who left Wuhan. In addition, several large cities in China also had higher proportions of travellers. For example, people who went to Chongqing, Beijing, Shanghai, Shenzhen and Guangzhou accounted for 1.27%, 0.86%, 0.66%, 0.50% and 0.50%, respectively, of the people who left Wuhan (Fig. 2 and Table 1).

**Table 1.** Proportion of the population travelling out of Wuhan to various cities

Province	City	Ranking	Percentage (%)
Hubei	Xiaogan	1	13.80
	Huanggang	2	13.04
	Jingzhou	3	6.54
	Ezhou	5	3.97
	Suizhou	9	3.21
	Xiangyang	6	3.93
	Huangshi	7	3.77
	Yichang	11	2.81
	Jiangmen	8	3.30
	Xianning	4	5.01
	Shiyan	13	1.86
	Xiantao	10	2.97
	Tianmen	12	2.08
	Enshi	14	1.81
	Qianjiang	17	1.14
Guangdong	Shenzhen	26	0.50
	Guangzhou	27	0.50
	Zhuhai	85	0.11
	Dongguan	69	0.13
	Foshan	84	0.11
	Huizhou	94	0.10
Henan	Xinyang	15	1.49
	Zhengzhou	23	0.59
	Nanyang	20	0.69
	Zhumadian	22	0.66
	Shangqiu	34	0.34
	Zhoukou	31	0.44
	Pingdingshan	68	0.14
	Xinxiang	56	0.17
	Anyang	66	0.15
	Xuchang	60	0.16
	Luohe	50	0.18
	Luoyang	48	0.19
	Kaifeng	67	0.14
Zhejiang	Wenzhou	42	0.21
	Hangzhou	39	0.25
	Ningbo	89	0.11
	Taizhou	87	0.11
	Jinhua	90	0.11
Hunan	Changsha	18	1.02
	Yueyang	25	0.52
	Shaoyang	49	0.19
	Changde	36	0.33
	Zhuzhou	51	0.18
	Loudi	55	0.17
	Yiyang	52	0.18
	Hengyang	40	0.24
	Yongzhou	77	0.12
	Huaihua	88	0.11
	Chenzhou	93	0.10
	Xiangtai	76	0.12
Anhui	Hefei	32	0.40
	Fuyang	33	0.35
	Bozhou	91	0.10
	Anqing	30	0.45
	Liuan	43	0.20
	Suzhou	95	0.10
	Wuhu	81	0.11
Jiangxi	Nanchang	28	0.48
	Shangrao	54	0.18
	Jiujiang	24	0.52
	Yichun	38	0.26
	Ganzhou	58	0.16
	Fuzhou	62	0.15
	Jian	70	0.13
Jiangsu	Nanjing	37	0.29
	Suzhou	47	0.19
	Xuzhou	65	0.15
	Wuxi	82	0.11
	Nantong	74	0.13
	Chouqing	16	1.27
Shandong	Qingdao	78	0.12
	Jinan	92	0.10
	Heze	63	0.15
Sichuan	Chendu	29	0.46
	Dazhou	80	0.11
Heilongjiang	Haerbin	61	0.16
	Beijing	19	0.86
	Shanghai	21	0.66
Heibei	Cangzhou	100	0.09
	Baoding	83	0.11
	Handan	59	0.16
	Shijiazhuang	53	0.18
	Xingtai	71	0.13
Fujian	Fuzhou	45	0.20
	Quanzhou	44	0.20
	Xiamen	57	0.16
Guangxi	Nanning	46	0.19
	Beihai	98	0.09
	Guilin	72	0.13
Shaanxi	Xian	35	0.34
	Ankang	97	0.10
Yunnan	Kunming	41	0.23
Hainan	Sanya	75	0.13
	Haikou	79	0.11
Guizhou	Guiyang	73	0.13
Shanxi	Taiyuan	86	0.11
	Tianjin	64	0.15
Liaoning	Shenyang	96	0.10
Gansu	Lanzhou	99	0.09

### Regression analysis of the proportion of migrants into each city and outbreak development

**Fig. 3.**
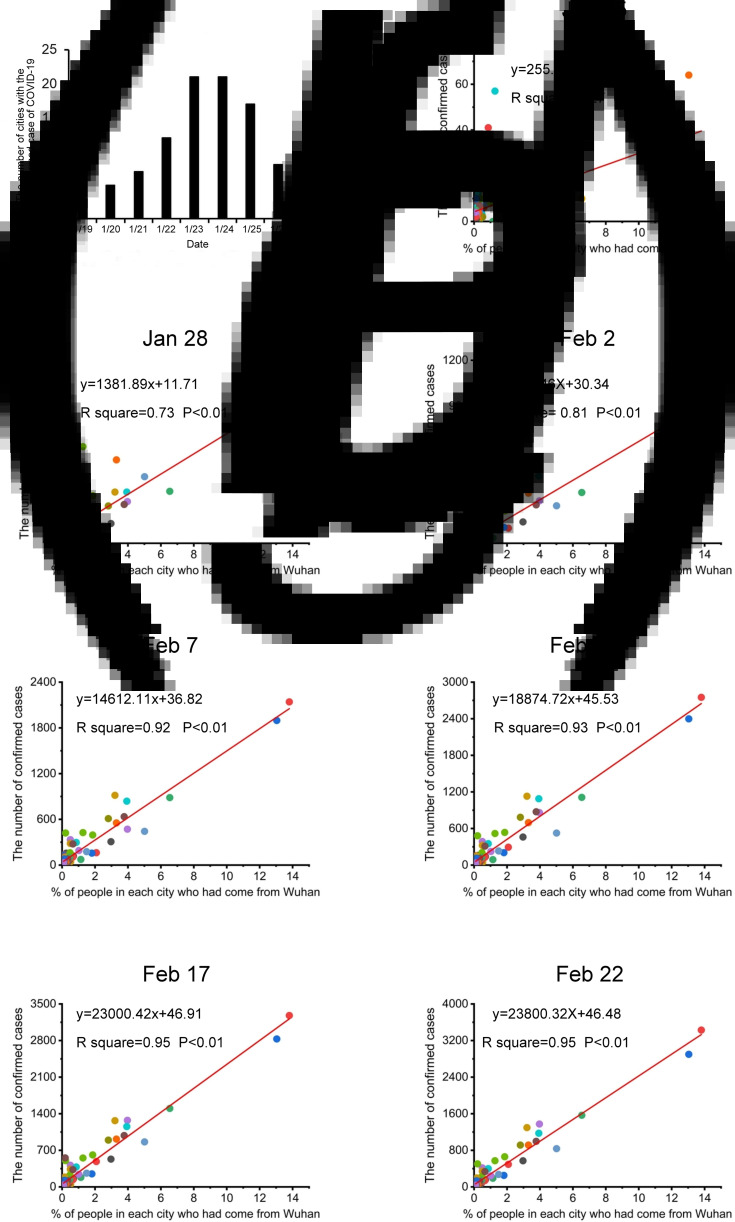
Correlation regression analysis between the proportion of the population travelling to destination cities from Wuhan and the cumulative confirmed number of COVID-19 cases in the destination cities. (a) Time when the first confirmed COVID-19 case occurred in the top 100 destination cities for people who left Wuhan. Correlation regression analysis between the proportion of people who travelled from Wuhan to a destination city and the cumulative confirmed cases of COVID-19 in the city on (b) 25 January, (c) 28 January, (d) 2 February, (e) 7 February, (f) 12 February, (g) 17 February and (h) 22 February (*n* = 100, * *P* < 0.05, ** *P* < 0.01).

On 19 January 2020, Shenzhen reported the first confirmed case of imported COVID-19, which was also the first confirmed case found outside Wuhan. Later, other cities also successively reported confirmed cases of COVID-19. By 27 January 2020, confirmed COVID-19 cases had appeared in the top 100 destination cities for people who travelled from Wuhan (Fig. 1). Figure 3a shows the number of cities with their first confirmed cases of COVID-19 from 19 January 2020 to 27 January 2020. Based on the correlation analysis, we found a significant positive correlation between the proportion of people in each city who had come from Wuhan from 10 January 2020 to 24 January 2020, and the cumulative number of confirmed cases of COVID-19 at the other time points (all *P* < 0.01) except on 23 and 24 January (all *P* > 0.05). Over time, the correlation coefficient *r* value increased gradually. The data are shown in Table 2.

**Table 2.** Correlation regression analysis between the proportion of the population travelling to destination cities and the cumulative confirmed number of COVID-19 cases

Date	*r*	*R^2^*	Constants	Slopes	s.e. of constants	s.e. of slopes	*F*	*P*
25 January	0.52	0.27	4.22	255.39	0.99	42.77	35.66	0.00
28 January	0.85	0.73	11.71	1381.89	1.97	84.99	264.37	0.00
2 February	0.92	0.84	30.34	6379.46	6.48	279.98	519.17	0.00
7 February	0.96	0.92	36.82	14 612.11	10.21	441.33	1096.24	0.00
12 February	0.96	0.93	45.63	18 874.72	12.22	528.00	1277.90	0.00
17 February	0.98	0.95	46.91	23 000.42	12.12	523.90	1927.40	0.00
22 February	0.97	0.95	46.48	23 800.32	12.75	551.23	1864.22	0.00

The regression analysis showed that there was a linear relationship between the proportion of people who left Wuhan for the destination cities and the cumulative confirmed number of COVID-19 cases at the other time points (all *P* < 0.01) except on 23 and 24 January (all *P* > 0.05) (Table 2). Over time, the *R*^2^ value increased gradually. Figure 3b–h shows the linear regression equation.

### Changes in the intensity of intracity travel and the growth rate of the number of confirmed COVID-19 cases during the outbreak

**Fig. 4.**
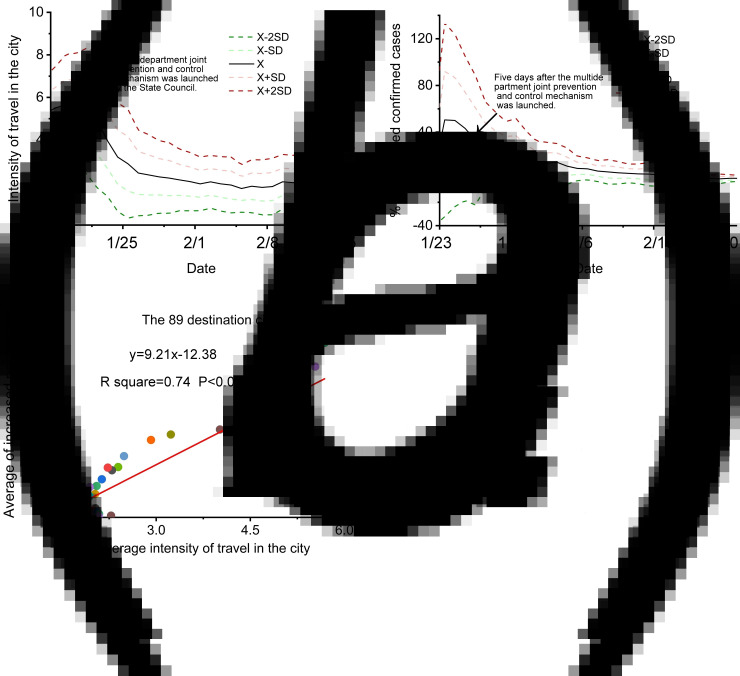
Changes in intracity travel intensity and the growth rate of the number of confirmed COVID-19 cases in these cities. (a) From 18 January to 17 February 2020, the average travel intensity of the top 100 destinations of people travelling out of Wuhan. (b) Changes in the average growth rate of the number of confirmed COVID-19 cases in 89 cities from 23 January to 22 February 2020 (11 cities were excluded from the study due to incomplete data of cumulative cured cases and cumulative deaths). (c) Correlation regression analysis between the changes in intracity travel intensity and growth rate of the number of confirmed COVID-19 cases in 89 cities from 23 January to 22 February 2020. The std. error of constants was 3.18. The std. error of slopes was 1.02 (* *P* < 0.05, ** *P* < 0.01).

To clarify the effect of changes in the intracity travel intensity of each city on the development of the outbreak, we calculated intracity travel intensity in 100 cities during the period from 18 January 2020 to 17 February 2020. The data provided by Baidu Maps Smarteye show that the average intracity travel intensities in the top 100 destination cities for people who left Wuhan on 18 January, 23 January, 28 January, 2 February, 7 February, 12 February and 17 February were 5.25 ± 0.87, 4.94 ± 1.59, 2.35 ± 0.95, 1.93 ± 0.63, 1.83 ± 0.62, 2.05 ± 0.66 and 2.28 ± 0.68, respectively. After the State Council launched prevention and control mechanisms on 22 January 2020, the intracity travel intensity of each city decreased by 60−70% (all *P* < 0.01; results are shown in Fig. 4a).

The average growth rates of the confirmed COVID-19 cases in the top 89 destination cities (11 cities were excluded from the study due to incomplete data of cumulative cured cases and cumulative deaths) were 6.67% ± 23.38%, 28.92% ± 18.50%, 13.97% ± 8.99%, 8.49% ± 4.71%, 4.24% ± 4.18%, 1.60% ± 2.39% and 0.48% ± 1.28% on 23 January, 28 January, 2 February, 7 February, 12 February, 17 February and 22 February, respectively. After 27 January 2020, the average growth rates of the number of confirmed COVID-19 cases in each city decreased significantly (all *P* < 0.01). The results are shown in Figure 4b.

### Correlation regression analysis between intracity travel intensity and the growth rate of the number of confirmed COVID-19 cases

The average incubation period of SARS-CoV-2 is five days. Thus, we explored the effect of the average intracity travel intensity in the 89 cities from 18 January to 17 February 2020, on the average growth rate of the number of confirmed COVID-19 cases in those cities from 23 January to 22 February 2020. The value of the correlation coefficient *r* between the average intracity travel intensity and the average growth rate of the number of confirmed COVID-19 cases in those cities was 0.86 (*P* < 0.001). Further regression analysis showed that the value of the regression coefficient *R*^2^ between intracity travel intensity and the growth rate of the number of confirmed COVID-19 cases in 89 cities was 0.74, with *P* < 0.01 indicating a significant linear relationship. The results are shown in Figure 4c.

## Discussion

SARS-CoV-2 is an infectious respiratory disease mainly spread by droplets. The population is generally susceptible to the disease. Clinically, fever, cough and fatigue are the main manifestations [16, 17]. Nucleic acid detection and imaging examination provide important clinical guidance for the diagnosis and treatment of patients [18]. Given the lack of effective drugs, clinical treatment still mainly involves symptomatic treatment and nutritional support [16, 17, 19]. Increases in population movement and enhanced mobility made it possible for SARS-CoV-2 to spread easily and quickly, making it difficult to control [20]. Moreover, the outbreak occurred in winter, which is a season with high incidence of various infectious respiratory diseases [21]. Affected by the Spring Festival and epidemic factors, a large number of latent virus carriers travelled from Wuhan to other cities, leading to the outbreak of COVID-19 throughout the country, affecting all 34 provincial regions [10]. In an attempt to prevent further dispersal of COVID-19, the Party Central Committee and the State Council launched a multidepartment joint prevention and control mechanism on 22 January 2020, and all transport was prohibited in and out of Wuhan city from 23 January 2020, followed by all Hubei Province one day later [12–14]. The implementation of prevention and control measures by Chinese governments at all levels helped slow the epidemic and prevent a second outbreak [10, 20].

The data on population movement provided by Baidu Maps Smarteye showed that from 10 January 2020 to 14 January 2020, people who left Wuhan for the top 100 destination cities accounted for 91.58% of the population travelling out of Wuhan. The destinations were mainly large and medium-sized cities in Hubei Province and other parts of China [11]. As of 23 February 2020, the cumulative confirmed cases of COVID-19 in these 100 cities accounted for 85.97% of the number of confirmed cases outside Wuhan [22]. The percentage was slightly lower than the proportion of people who came from Wuhan. This may be related to the role of these cities as transportation hubs − that is, although the first destination of people who left Wuhan was the abovementioned 100 large and medium-sized cities, the final destination was not one of those cities [23]. Rather, people travelled through these cities to other small and medium-sized cities. This led to the abovementioned difference between the proportion of people who left Wuhan and the cumulative number of confirmed cases. The correlation regression analysis showed that the proportion of people who travelled from Wuhan to the top 100 cities after 24 January was closely correlated with the cumulative confirmed cases of COVID-19 in each city, showing a significant linear relationship. The fact that there was no correlation on 23 and 24 January could be related to the fact that some patients were in the incubation period and had not yet developed the disease.

The basic principles of infectious disease prevention and control include controlling the source of infection, blocking the route of transmission and protecting susceptible individuals [24]. Vaccines are an important means of protecting susceptible people. Since a vaccine for SARS-CoV-2 has not yet been developed, the current focus is mainly on controlling the source of infection and blocking the transmission route [24, 25]. The data provided by Baidu Maps Smarteye show that since the implementation of national prevention and control measures (22 January), the intensity of intracity travel in the abovementioned 100 cities decreased by 60−70%, and in Wuhan, it decreased by more than 83%, representing 20−40% of the average level of travel intensity in other cities during the same period [11]. Through the analysis of average growth rates of confirmed COVID-19 cases, we found that it decreased significantly from 27 January 2020. Due to the various prevention and control measures, as of 23 February, 79 of the abovementioned 100 cities had no new cases, thus slowing the epidemic and demonstrating the effectiveness of strict restrictions on population movement [22]. Such measures have also prevented a second wave of COVID-19 outbreaks. The correlation regression analysis results showed that the intracity travel intensity of a city on the *n*th day was positively related to the growth rate of the number of confirmed COVID-19 cases on the *n* + 5th day in that city, showing a significant linear relationship.

In this study, it is worth noting that from a developer's perspective, the number of Baidu Maps open-platform developers exceeds 1.65 million, providing services for more than 650 000 PPS and websites from a user's perspective. Baidu positioning services respond to global location service requests more than 120 billion times per day. This large amount of location request data provided accurate data to support tracking of the movement of Wuhan's population [26].

The correlation regression analysis of the proportion of people leaving Wuhan for destination cities on the eve of the outbreak, the intensity of intracity travel, and the development of the outbreak in the destination cities showed that the proportion of people who travelled from Wuhan to the top 100 cities after 24 January was closely correlated with the cumulative confirmed cases of COVID-19 in each city, showing a significant linear relationship. Thus, the effective implementation of prevention and control measures, such as restricting the movement of people, can significantly curb the development of an outbreak, help control the source of infection and block the route of transmission. It should be noted that since this study only focused on the proportion of Wuhan's population moving to destination cities and the travel intensity in each destination city, it did not account for population flows between other cities and differences in cities' population densities, climate, medical capacity and implementation of control measures. The study therefore has certain limitations. It does, however, provide a unique method, allowing us to observe a possible potential variable and establish a theoretical scientific foundation for formulating prevention and control strategies and intervention techniques.

## Data Availability

The data for the study is available by contacting the corresponding author
